# Semi-automated quantification of tricuspid valve dynamics and structure in tetralogy of Fallot and hypoplastic left heart syndrome using three-dimensional echocardiography

**DOI:** 10.1186/s44156-023-00023-y

**Published:** 2023-07-06

**Authors:** Vivek Jani, Ling Li, Mary Craft, Federico Veronesi, Nee Khoo, David Danford, Denisa Muraru, Shelby Kutty

**Affiliations:** 1 Taussig Heart Center, Department of Pediatrics, Johns Hopkins Hospital, Baltimore, MD USA; 2 University of Nebraska Medical Center, Children’s Hospital and Medical Center, Omaha, NE USA; 3 Department of Electrical, Electronic and Information Engineering, University of Bologna, Bologna, Italy; 4 Division of Cardiology, Department of Pediatrics, Stollery Children’s Hospital, University of Alberta, Edmonton, AB Canada; 5 Department of Medicine and Surgery, University of Milano-Bicocca, Milan, Italy; 6 Department of Cardiology, Istituto Auxologico Italiano, IRCCS, Milan, Italy

**Keywords:** Tricuspid valve, 3D echocardiography, Repaired tetralogy of Fallot, Hypoplastic left heart syndrome

## Abstract

Anomalies of the tricuspid valve (TV) are associated with worsened prognosis in congenital heart disease (CHD). Here, we present a descriptive study examining changes in TV morphology in two CHD conditions—repaired tetralogy of Fallot (rTOF) and hypoplastic left heart syndrome (HLSH), using three-dimensional echocardiography. Full volume acquisitions of the TV and right ventricle (RV) were performed from an RV-focused apical view using ECG gating over 2–5 consecutive cardiac cycles using 3D echocardiography, from which TV annulus and leaflet parameters were quantified. A total of 40 rTOF patients (age 14 ± 9.8 years), 40 HLHS patients (age1.0 ± 1.5 years) and 80 age and gender matched controls were included. Among leaflet parameters, antero-posterior and posterior-septal TV coaptation heights were smaller in rTOF (p < 0.001) vs. control. Conversely, only the short-axis TV height was different in HLHS vs. controls (HLHS 1.6 ± 0.4 cm vs. control 1.4 ± 0.3 cm). TV leaflet parameters tended to be larger in HLHS, while leaflet coaptation distances were similar between groups. We demonstrate that 3D echocardiography for assessment of the TV is feasible in rTOF and HLHS patients and identifies unique differences in TV morphology. Future studies should clarify the clinical significance of TV morphology in these patient populations.

## Introduction

Tricuspid regurgitation (TR) and other abnormalities of tricuspid valve (TV) function are associated with increased mortality in congenital heart disease (CHD) [1, 2]. The concept of abnormal TV function irrespective of TR is acknowledged in current guidelines for TV replacement in patients undergoing left-heart surgery [3, 4]. Tricuspid regurgitation and TV dysfunction are known to correlate with adverse clinical outcomes in both hypoplastic left heart syndrome (HLHS) [1] and repaired tetralogy of Fallot (rTOF) [2]. Previously, we reported gross structural changes in TV morphology in HLHS that can be quantified with 3D echocardiography of the tricuspid valve [5, 6]. Here, we present a descriptive study examining changes in the TV in rTOF, a condition with RV remodeling from chronic exposure to upstream afterload, hypothesizing that advanced imaging of the TV using 3D echocardiography may provide insight into TV structure and function. We subsequently compare these changes to HLHS, a congenital condition with known gross anatomical changes to the TV.

## Methods

**Fig. 1:**
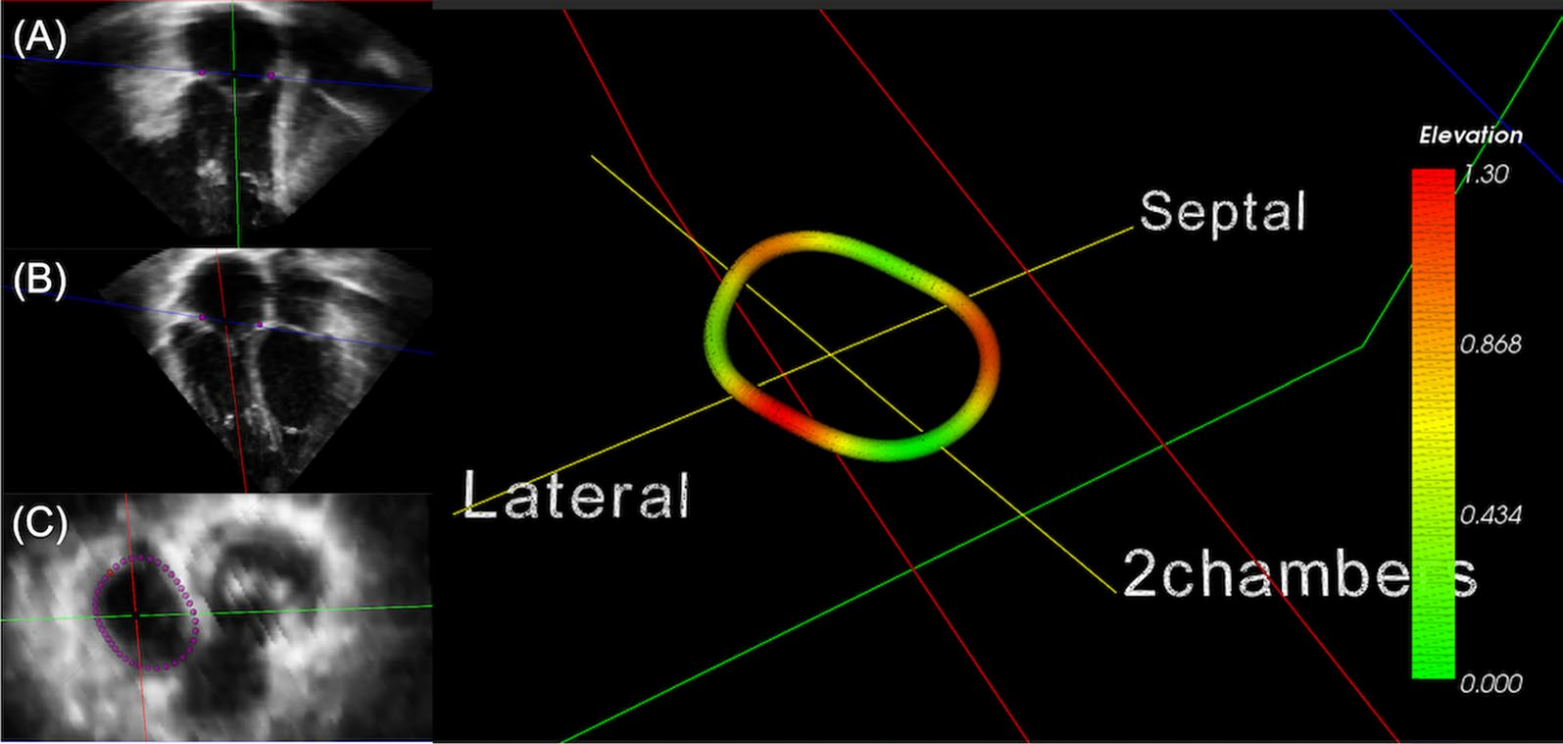
Volume acquisition of the tricuspid valve. Identification of tricuspid valve from (**A**) two and (**B**) four chamber views. **C** Estimated tricuspid valve contour. Shown on the right are tricuspid valve annulus and leaflet parameter measurements

This study was approved by the University of Nebraska Medical Center institutional review board. Informed consent was obtained from each patient, and the study protocol conforms to the ethical guidelines of the 1975 Declaration of Helsinki as reflected in a priori approval by the institution’s human research committee. Participants underwent prospective 3DE using the iE33 system (Philips) or Vivid E9 (GE). Full volume acquisitions of the TV and right ventricle (RV) were performed from an RV-focused apical view using ECG gating over 2–5 consecutive cardiac cycles. Annular [area, perimeter, circularity, dimensions at late diastole, inter-commissural distances (length of the annular chord between commissures)] and leaflet parameters [tenting height, tenting volume, area (anterior, posterior, septal, total), coaptation heights [anteroseptal (AS), posteroseptal (PS), anteroposterior (AP)] were measured using custom software (TomTec Inc, Unterschleissheim Germany), as shown in Fig. 1 [7]. A single board-certified echocardiographer identified the optimal 4-chamber and orthogonal view and manually initialized the tricuspid annulus (TA) by marking the TV leaflet hinge points in 8 planes rotated about the center (Fig. 1). The software resampled 80 points along the entire TA and tracked them throughout the cardiac cycle to determine TV parameters. Two-tailed independent sample Student t-test was used to compare TA parameters between the rTOF group and its controls, and the HLHS group and its controls. Each population had its own age-matched control group to account for differences in age and pathophysiology between groups. Exclusion criteria for rTOF patients include prior pulmonary valve replacement or tricuspid valve intervention.

## Results

A total of 40 rTOF subjects (23:17 M:F, age 14 ± 9.8 years, BSA 1.3 ± 0.5 m^2^), 40 HLHS subjects (23:17 M:F, age 1.0 ± 1.5 years, BSA 0.4 ± 0.2 m^2^), and 80 age and gender matched controls (40 matched to the rTOF group and 40 to the HLHS group) were included in this study. Among HLHS patients, 7 were pre-Norwood, 19 pre-Glenn, 10 pre-Fontan and 4 post-Fontan. Patient characteristics and TV annulus and leaflet parameters from 3DE are summarized in Table 1. Tricuspid valve annulus parameters in rTOF did not differ significantly from controls. Among leaflet parameters, however, AP and PS coaptation heights were significantly smaller in rTOF (p < 0.001) than in controls, whereas individual and total leaflet areas were similar to controls. Among HLHS patients, only short-axis TV height was different from controls (1.6 ± 0.4 cm in HLHS vs. 1.4 ± 0.3 cm in controls; p < 0.05). Tricuspid valve leaflet parameters tended to be larger in HLHS, while leaflet coaptation distances were similar between groups.

**Table 1:** Tricuspid valve annulus and leaflet parameters in tetralogy of Fallot and hypoplastic left heart syndrome

	TOF	TOF Controls	HLHS	HLHS controls
Age	14.7 ± 9.8	14.8 ± 9.8	1.0 ± 1.5	1.0 ± 1.5
BSA	1.3 ± 0.54	1.4 ± 0.60	0.35 ± 0.17	0.42 ± 0.24
Annulus parameter				
Area (cm^2^)	6.5 ± 3.7	7.7 ± 3.9	2.5 ± 1.2	2.1 ± 0.9
Perimeter (cm)	8.9 ± 2.8	9.8 ± 2.5	5.7 ± 1.3	5.3 ± 1.1
Long axis length (cm)	3.1 ± 1.0	3.4 ± 0.8	2.0 ± 0.5	1.8 ± 0.4
Short axis length (cm)	2.5 ± 0.9	2.8 ± 0.9	1.6 ± 0.4	1.4 ± 0.3
Circularity	0.82 ± 0.1	0.83 ± 0.1	0.82 ± 0.1	0.80 ± 0.1
Intercommissural distance anterior (mm)	24.5 ± 9.0	24.5 ± 7.4	14.9 ± 4.3	14.1 ± 3.3
Intercommissural distance posterior (mm)	20.8 ± 7.0	25.0 ± 7.1	14.1 ± 5.1	12.4 ± 3.5
Intercommissural distance septal (mm)	24.2 ± 8.7	27.8 ± 8.1	14.9 ± 3.8	13.8 ± 4.2
Leaflet parameters				
Tenting height (cm)	17.3 ± 5.7	20.7 ± 5.0	11.0 ± 3.5	11.0 ± 3.5
Tenting volume (ml)	2.5 ± 1.9	2.6 ± 1.8	0.9 ± 0.6*	0.5 ± 0.3*
Total leaflet area (cm^2^)	7.7 ± 4.0	7.7 ± 3.5	4.1 ± 1.9*	2.6 ± 1.1*
Area anterior (cm^2^)	2.4 ± 1.4	2.0 ± 1.0	1.5 ± 1.1*	0.8 ± 0.4*
Area posterior (cm^2^)	2.1 ± 1.1	2.5 ± 1.4	1.1 ± 0.7*	0.8 ± 0.5*
Area septal (cm^2^)	2.8 ± 1.8	2.8 ± 1.5	1.3 ± 0.7*	0.8 ± 0.5*
Coaptation height AS (mm)	17.8 ± 5.7*	22.6 ± 6.5*	10.5 ± 3.3	10.8 ± 3.6
Coaptation height PS (cm)	16.7 ± 5.6*	22.2 ± 6.0*	10.3 ± 3.1	9.5 ± 3.9
Coaptation height AP (mm)	19.9 ± 6.7	22.8 ± 6.8	11.2 ± 4.1	10.4 ± 4.1

*p < 0.001 compared to respective controls; *TOF* tetralogy of Fallot, *HLHS* Hypoplastic Left Heart Syndrome, *BSA* body surface area, *AP* anteroposterior, *AS* anteroseptal, *AP* anteroposterior

## Discussion

Principally, we found: (1) As demonstrated previously in HLHS [5], 3D echocardiography of the TV is feasible in rTOF. (2) In HLHS, TV leaflets were uniformly larger compared to controls; this leaflet-annular disproportion results in greater tenting volumes in HLHS, while maintaining normal leaflet coaptation distances; (3) Unlike in HLHS where gross structural abnormalities in the TV are present, 3D echocardiography identified significant differences in TV structure and function in rTOF. Specifically, we see that the TV annulus was only mildly dilated, and septal leaflet coaptation heights were short.

In rTOF, the observed short septal leaflet coaptation height likely reflects anatomic changes due to the ventricular septal patch, though the relation between septal leaflet coaptation and the development of TR is less clear. It is well established that RV dilatation secondary to volume overload contributes to the development of TR. Tricuspid annulus size is the strongest predictor of TR, but coaptation distance is also significant[5, 7]. These changes may reflect TV maladaptation secondary to surgical palliation, similar to what has been observed for the mitral valve [5]. In HLHS, the TV adapts to interstage stress by increasing leaflet size out of proportion to growth, whereas changes in TV morphology in rTOF result from chronic volume overload [5, 8]. Our study has limitations. Within the HLHS cohort, patients at all stages of palliation and different anatomical subtypes were included, each of which with different loading conditions that impact TV function [5, 6, 9]. Moreover, rTOF patients have different degree of RV volume overload, depending on their age and timing from surgery, which influences TV function and is not addressed here. The relationship between septal leaflet tethering and TV regurgitation is unclear and requires further investigation. Moreover, the clinical significance of these findings requires longitudinal follow-up and is left to future studies. It remains possible that the changes are adaptation to altered loading conditions, and this too is left to future studies.

In summary, despite the lack of gross structural abnormalities as observed in HLHS, 3D echocardiography of the TV can provide meaningful structural and functional differences in rTOF. Specifically, we confirmed prior studies identifying leaflet-annular disproportion in HLHS and identify a unique signature of septal leaflet coaptation in rTOF that is not observed in HLHS. These results may have implications for understanding TV maladaptation to chronic changes in loading conditions in CHD, for timing surgical management, and potential for application to 3D printing of TV in congenital heart disease [10]. Future studies should clarify the clinical significance of TV morphology in these congenital populations and evaluate its value for guiding the timing and specific nature of surgical interventions.

## Data Availability

Data will be made available upon reasonable request.

## References

[1] Newburger JW, Sleeper LA, Frommelt PC, Pearson GD, Mahle WT, Chen S, Dunbar-Masterson C, Mital S, Williams IA, Ghanayem NS (2014). Transplantation-free survival and interventions at 3 years in the single ventricle reconstruction trial. Circulation.

[2] Bokma JP, Winter MM, Oosterhof T, Vliegen HW, van Dijk AP, Hazekamp MG, Koolbergen DR, Groenink M, Mulder BJ, Bouma BJ (2015). Severe tricuspid regurgitation is predictive for adverse events in tetralogy of Fallot. Heart.

[3] Dreyfus GD, Corbi PJ, Chan KJ, Bahrami T (2005). Secondary tricuspid regurgitation or dilatation: which should be the criteria for surgical repair?. Ann Thorac Surg.

[4] Hahn RT, Weckbach LT, Noack T, Hamid N, Kitamura M, Bae R, Lurz P, Kodali SK, Sorajja P, Hausleiter J (2021). Proposal for a standard echocardiographic tricuspid valve nomenclature. Cardiovasc Imaging.

[5] Colen T, Kutty S, Thompson RB, Tham E, Mackie AS, Li L, Truong DT, Maruyama M, Smallhorn JF, Khoo NS (2018). Tricuspid valve adaptation during the first interstage period in hypoplastic left heart syndrome. J Am Soc Echocardiogr.

[6] Kutty S, Colen T, Thompson RB, Tham E, Li L, Vijarnsorn C, Polak A, Truong DT, Danford DA, Smallhorn JF (2014). Tricuspid regurgitation in hypoplastic left heart syndrome: mechanistic insights from 3-dimensional echocardiography and relationship with outcomes. Circ Cardiovasc Imaging.

[7] Addetia K, Muraru D, Veronesi F, Jenei C, Cavalli G, Besser SA, Mor-Avi V, Lang RM, Badano LP (2019). 3-Dimensional echocardiographic analysis of the tricuspid annulus provides new insights into tricuspid valve geometry and dynamics. JACC Cardiovasc Imaging.

[8] Benedetto U, Melina G, Angeloni E, Refice S, Roscitano A, Comito C, Sinatra R (2012). Prophylactic tricuspid annuloplasty in patients with dilated tricuspid annulus undergoing mitral valve surgery. J Thorac Cardiovasc Surg.

[9] Nguyen AV, Lasso A, Nam HH, Faerber J, Aly AH, Pouch AM, Scanlan AB, McGowan FX, Mercer-Rosa L, Cohen MS (2019). Dynamic three-dimensional geometry of the tricuspid valve annulus in hypoplastic left heart syndrome with a Fontan circulation. J Am Soc Echocardiogr.

[10] Muraru D, Veronesi F, Maddalozzo A, Dequal D, Frajhof L, Rabischoffsky A, Iliceto S, Badano LP (2017). 3D printing of normal and pathologic tricuspid valves from transthoracic 3D echocardiography data sets. Eur Heart J Cardiovasc Imaging.

